# Real-time rate of penetration prediction for motorized bottom hole assembly using machine learning methods

**DOI:** 10.1038/s41598-023-41782-2

**Published:** 2023-09-03

**Authors:** Amir Shokry, Salaheldin Elkatatny, Abdulazeez Abdulraheem

**Affiliations:** Department of Petroleum Engineering, College of Petroleum Engineering & Geosciences, King Fahd University of Petroleum and Minerals, 31261 Dhahran, Saudi Arabia

**Keywords:** Solid Earth sciences, Engineering

## Abstract

Drilling rate of penetration (ROP) is one of the most important factors that have their significant effect on the drilling operation economically and efficiently. Motorized bottom hole assembly (BHA) has different applications that are not limited to achieve the required directional work but also it could be used for drilling optimization to enhance the ROP and mitigate the downhole vibration. Previous work has been done to predict ROP for rotary BHA and for rotary steerable system BHA; however, limited studies considered to predict the ROP for motorized BHA. In the present study, two artificial intelligence techniques were applied including artificial neural network and adaptive neurofuzzy inference system for ROP prediction for motorized assembly in the rotary mode based on surface drilling parameters, motor downhole output parameters besides mud parameters. This new robust model was trained and tested to accurately predict the ROP with more than 5800 data set with a 70/30 data ratio for training and testing respectively. The accuracy of developed models was evaluated in terms of average absolute percentage error, root mean square error, and correlation coefficient (R). The obtained results confirmed that both models were capable of predicting the motorized BHA ROP on Real-time. Based on the proposed model, the drilling parameters could be optimized to achieve maximum motorized BHA ROP. Achieving maximum ROP will help to reduce the overall drilling cost and as well minimize the open hole exposure time. The proposed model could be considered as a robust tool for evaluating the motorized BHA performance against the different BHA driving mechanisms which have their well-established models.

## Introduction

Motorized BHA plays an important role nowadays in delivering deviated wells or even keeping the verticality in other wells when combining with the bent housing ^1^. It could be also used with zero bent housing to deliver more energy to the drilling bit to enhance the ROP ^2^. The concept of use the mud motor to achieve more bit RPM is not new. In 1873, the turbodrill mud motor patent was issued ^3^. Down hole mud motors are powered by mud flow. The two major types of down hole motor are: the Turbine mud motor which is basically a centrifugal or axial pump and the Positive Displacement Motor (PDM) ^4^. Both types are driven by the circulating fluids. They are both equipped with rotating and stationary sections which makes it possible to drill without rotating the drill string ^5^. PDM is commonly used more than the turbine mud motor and it’s mainly made of several sections; the bypass valve or dump valve, the motor section, the universal joint or connecting rod assembly and the bearing section with drive sub. The bypass valve or dump valve section is optional part ^6^. PDM operating characteristics are very simple as the motor torque is directly proportional to the motor differential pressure and the motor RPM is directly proportional to the flow rate ^7^. The PDM efficiency could be expressed by the mechanical power output divided by hydraulic power input as per the below Equations^8^;

1 $$ {\text{PDM}}\,{\text{ hydraulic}}\,{\text{ power}}\,{\text{ input }}\,\left( {{\text{HP}}} \right) = \frac{P \times Q}{{1714}} $$

2 $$ {\text{PDM}}\,{\text{ mechanical }}\,{\text{power}}\,{\text{ output }}\,\left( {{\text{HP}}} \right) = \frac{T \times N}{{5252}} $$

3 $$ {\text{PDM }}\,{\text{efficiency}} = \frac{PDM\, mechanical \,power\, output }{{PDM\, hydraulic \,power\, input \,252}} $$

where P = The differential pressure through the motor power section (psi), Q = The flow rate (GPM), T = The motor output torque (lbs-ft), N = The bit speed (RPM).

There are two modes when working with the PDM motor; the rotary mode and the sliding mode. The rotary mode usually exhibits an azimuth hold tendency and in this mode the bit RPM will be higher than the surface RPM by the motor RPM. The sliding mode which is known as the oriented mode will exhibit a change in the azimuth value and the bit RPM in this case will be the motor RPM only as the surface RPM will be zero. A slight hole enlargement will happen with the rotary mode based on the value of the bent housing.

Drilling parameters and mud parameters have a direct effect on the ROP and there are different models that had been delivered to describe that effect ^9^. There are some mathematical analytical equations and empirical correlations that describe that relationship but those models are unreliable for all applications due to the complexity of the drilling process and the different factors and BHA types that could affect the ROP ^10^. Different models were established to consider the rotary BHA ROP such as Maurer who derived his model for a rotary BHA with a tri-cone bit assuming that the cutting are removed with each bit revolution ^11^. Bingham derived his model for rotary BHA ROP as a function of the applied WOB and RPM only based on laboratory analysis using a tri-cone bit also ^12^. Teale derived his stationary model to relate the ROP with applied WOB, RPM and produced torque through the concept of mechanical specific energy (MSE) ^13^. Warren developed his model to calculate the rotary BHA ROP with a tri-cone bit from the cleaning model concept and that model considered the effect of the overbalance effect on reducing the ROP ^14^. Hareland model was mainly based on Warren model with the consideration of the effect of bit wear on the ROP ^15^. Rumzan and Schmitt considered the effect of rock hardness on ROP in terms of depth as the depth increases the rock hardness increases ^16^. Teal model was the base for Armenta model which considered the effect of the fluid hydraulics on the ROP ^17^. Shokry et al. developed their model for two cutting structure BHA based on the MSE concept when they considered NOV eccentric dog-leg reamer ^18^.

The concept of Mechanical Specific Energy (MSE) was firstly presented by Teale in 1965 who defined it as the amount of work required to remove a unit volume of the rock ^13^. Different drilling bits performance can be evaluated with the help of applying MSE concept that leads to a significant improvement in the drilling efficiency by enhancing the ROP ^19,20^. Moreover, MSE has different useful applications such as diagnose different drilling problems and take appropriate steps to correct them through a continuous monitoring of MSE ^20,21^. MSE surveillance could be used to enhance the ROP by identifying the bit whirl and drill string vibrations then setting the optimum drilling parameters to reduce the level of the vibration severity ^22,23^. Formation lithology could be predicted in a real-time mode and the formation tops could be accurately determined from the MSE profile changes ^24^. Some MSE based models have been presented in the literature for different BHA driving mechanisms including rotary BHA, Rotary Steerable System (RSS) BHA and motor BHA as well based on the evaluation of key MSE models and the analysis on PDM performance, meanwhile methods for drilling performance prediction and optimization based on MSE technologies are presented ^25^.

Formation characteristics and the rock mineral composition have a direct effect on the ROP. The most important formation characteristics that affects the ROP are the formation elastic limit and the ultimate strength ^26^. The degree of the formation strength is usually determined using Mohr failure criterion. The mineral composition greatly affects the ROP and the bit life. Rocks containing abrasive minerals can lead to rapid wear of the drill bit teeth. Bit balling usually happens through the formation that has gummy clays ^27^. Other formation properties can also affect the ROP such as formation permeability. In high permeability rocks, the drilling fluid filtrate can invade quickly into the formation leading to differential pressure equalization across the drilled chips that enhance the ROP ^28^.

Recently artificial intelligence (AI) and machine learning have a wide range of applications covering different aspects in the oil and gas industry ^29–34^. Some machine learning models have been developed to predict ROP for rotary BHA and RSS BHA for vertical and deviated wells based on the surface drilling parameters and also the mud parameters in some models. Jahanbakhshi et al. used a wide range of field data with different lithology and used the surface drilling data as input data to develop his ANN model to predict the ROP ^35^. Kowakwi et al. included the effect of the overbalance degree and intoduce some modifications to Jahanbakhshi et al. model that improved the ROP prediction as he considered the bit wear and the hydraulics effect. Elkattattny et al. were able to predict rotary ROP based on the surface drilling parameters and some mud parameters as well for a vertical well using ANN techniques ^36^. Ahmed et al. included the mud parameters with surface drilling parameters and constructed SVM model to predict the ROP using 10 input features and he showed that SVM outperformed the other well-known theoretical equations with high margin ^37^. Al-AbdulJabbar et al.^38^ clustered the formation into different groups for better ROP prediction using the rock confined compressive strength (CCS), mud properties and surface drilling parameters. Amadi et al.^39^ developed a model to predict ROP from some derived variables from the drilling parameters using ANN for RSS system considering the effect of sticky slip vibration on ROP reduction. David Duru et al. tried to optimize the previous conventional models for ROP prediction though the genetic algorithm technique (GA) with R^2^ of 0.98 after the GA technique ^40^. Hongbao Zhang et al.^41^ used the data obtained from 82 wells and developed a ML model to predict ROP considering the well path, the rock mechanical properties, bit characteristics and formation heterogeneity index. Fan et al.^42^ developed different ML models based on physical mechanism constraints using data set from four wells in the same field. It’s clear from the literature that considered work had been delivered to predict the ROP from theoretical equations or using ML models for rotary BHA and RSS as well considering different factors that could affect the ROP and few models considered the effect of downhole motors in increasing the downhole RPM and introduce downhole torque.

The main goal of this study is to develop AI models that can accurately predict the ROP for a motorized BHA based on the surface drilling parameters, mud motor output, mud weight and temperature as well which are recorded in Real-time through a sensor on the mud return line. Six wells from the western desert of Egypt were used with a total of 5800 intermediate hole data points to generate and evaluate AI models using ANN and ANFIS.

## Methodology

**Figure 1:**
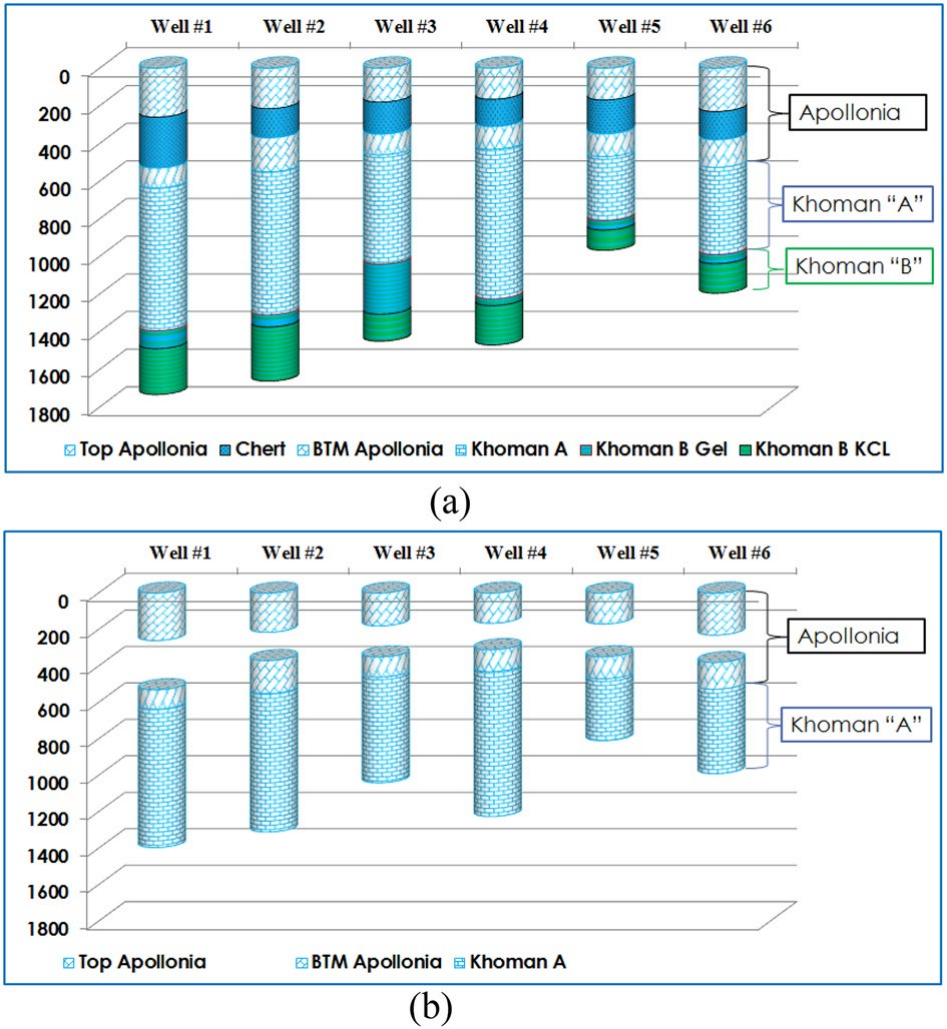
Formation Prognosis map for the six wells used in this study (**a**) complete wells with all lithology (**b**) after eliminating the Shale and Chert intervals.

Field measurements for 12½” intermediate hole were collected from six wells in the Egyptian western desert passing through Apollonia, Khoman”A” and Khoman”B” formations (Fig. 1a). Those six wells obtained from two nearby fields to eliminate the effect of formation characteristics change. The intermediate hole in those two fields could be delivered with different BHA such as rotary BHA, Motorized BHA and RSS BHA based on the well objective, well trajectory, mud parameters, drilling bit compatibility, formation prognosis and other different parameters. The drive mechanism applied in the selected six wells was the motorized BHA with $$9{\raise0.7ex\hbox{$5$} \!\mathord{\left/ {\vphantom {5 8}}\right.\kern-0pt} \!\lower0.7ex\hbox{$8$}}$$ “Geoforce motor (6/7 lobes, 3.5 stages, 0.11 RPG, with1.15 BH, $$12{\raise0.7ex\hbox{$1$} \!\mathord{\left/ {\vphantom {1 8}}\right.\kern-0pt} \!\lower0.7ex\hbox{$8$}}$$” sleeve). The drilling fluid used in the selected wells was water-based mud and the BHA accessories were identical. The intermediate section in the selected wells was delivered using Polycrystaline Diamond Compact (PDC) bit with six blades and 16 mm cutter size. The bit nozzles total flow area (TFA) was obtained the same in all wells at 0.902 in^2^. Those wells were delivered with the same drilling unit and the same drilling team which means all the factors that could affect the ROP are identical except for the applied drilling parameters and the motor output.

The obtained data include surface drilling parameters and mud parameters recorded at each one meter. The drilling bit RPM is not equal to the surface RPM as the mud motor will produce extra downhole RPM so the bit RPM at this case will be equal to the surface RPM and the motor RPM. The positive displacement motor RPM is dependent on the flow rate and the motor output so we could calculate the motor RPM (Eq. 4) then add it to the surface RPM to have the bit RPM (Eq. 5).

4 $$ {\text{Motor }}\,{\text{RPM }} = {\text{ Flow}}\,{\text{ rate }}\left( {{\text{GPM}}} \right) \, \times {\text{ Motor}}\,{\text{ output }}\left( {{\text{RPG}}} \right) $$

5 $$ {\text{Drill}}\,{\text{ Bit}}\,{\text{ RPM }} = {\text{ Surface}}\,{\text{ RPM }} + {\text{ Motor }}\,{\text{RPM}} $$

The bit torque in the motorized BHA is directly proportional to the motor differential pressure (ΔP_MTR_) so it’s important to include the motor differential pressure in the model input data. The recorded standpipe pressure is obtained while the bit on bottom as it’s recorded on the basis of depth increase. This value is representing the total system friction pressure plus the motor differential pressure so the motor differential pressure could be obtained from the difference between the on bottom stand pipe pressure (SPP_on BTM_) and the off bottom stand pipe pressure (SPP_off BTM_), the two recorded values should be at the same flow rate to keep the friction term constant in both terms and the difference will be the motor differential pressure (Eq. 6).

6 $$ \Delta {\text{P}}_{{{\text{MTR}}}} = {\text{ SPP}}_{{{\text{on}}\,{\text{ BTM}}}} - {\text{ SPP}}_{{{\text{off}}\,{\text{ BTM}}}} $$

After obtaining the motor operating parameters and perform the required data filtering and cleaning, two AI models were trained and validated based on 70% random selection of the data then the remaining 30% of the data was used for testing the trained model.

The trained model was used to predict the ROP for a new unseen well to confirm the model validity and capability for prediction for new wells.

### Data description

The obtained surface drilling parameters included weight on bit (WOB), surface revolution per minute (RPM), surface torque (TQ), standpipe pressure (SPP), and flow in rate (GPM) in addition to some mud parameters such as the mud weight (M.Wt) and the mud temperature (M.T) which are recorded Real-time using sensors on the return flow line with the corresponding rate of penetration (ROP) values. Motor performance parameters were obtained using the previous presented equations (Eqs. 4–6) to be within the model input data.

As the quality of the data is a main key factor for the AI model to catch the physical phenomena ^43^. As the formation characteristics and the rock mineral composition have a direct effect on the ROP, the data was filtered to represent lime stone lithology and eliminate the chert and shale intervals to help the model to predict better as shown in Fig. 1b. The data filtration and zonation process was performed based on the mud log to remove any non-Lime stone formation. The data is also filtered to remove any sliding intervals and keep only the rotary intervals. After this, data cleaning process was based on Z-score method for any values beyond three standard deviation from the sample mean. Then the dataset was cleaned for any missing data, duplicated values or sensors malfunction reading and those values which are unreasonable from the engineering concept. A total of 5800 data points obtained after cleaning and filtration process from those six wells.

**Figure 2:**
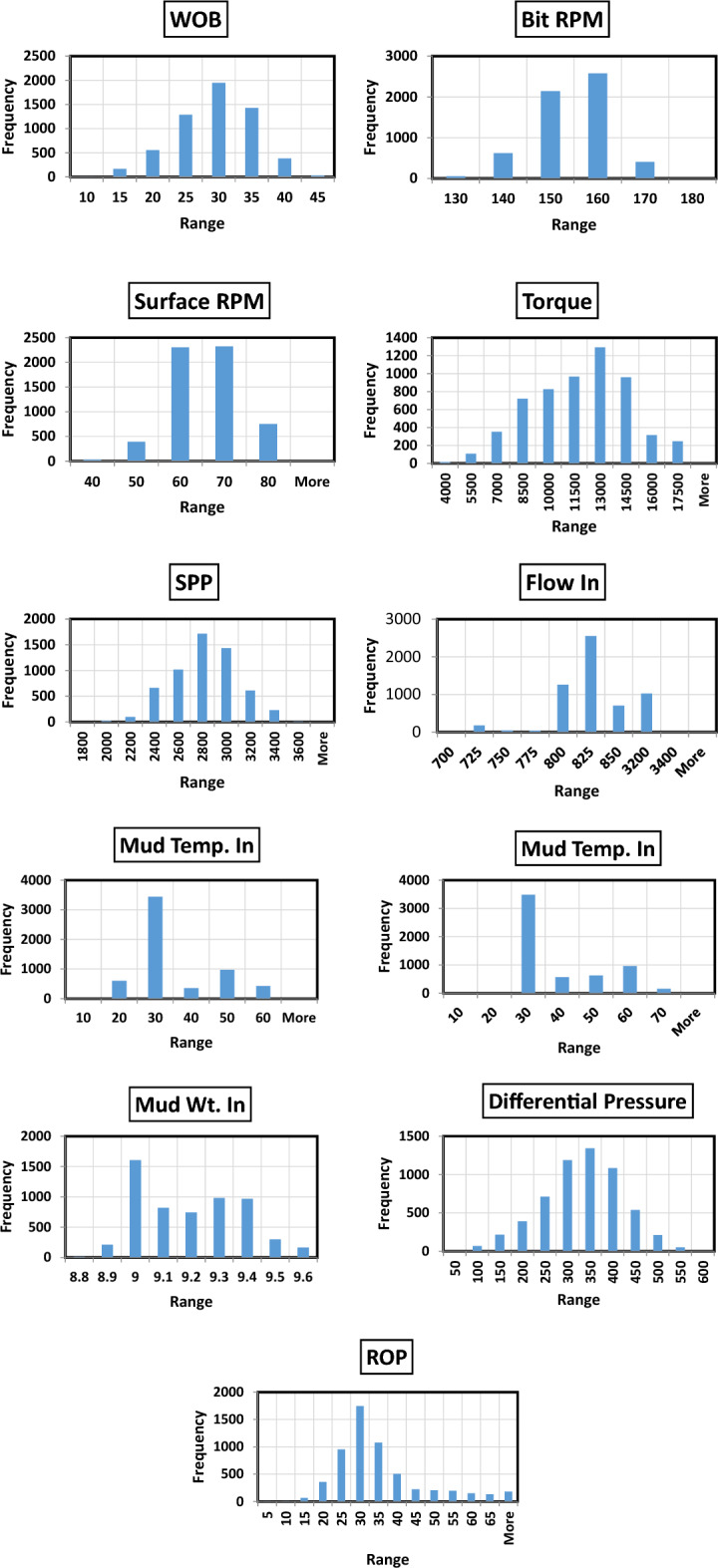
Distribution histogram for each parameter in the dataset after cleaning and filtering.

**Figure 3:**
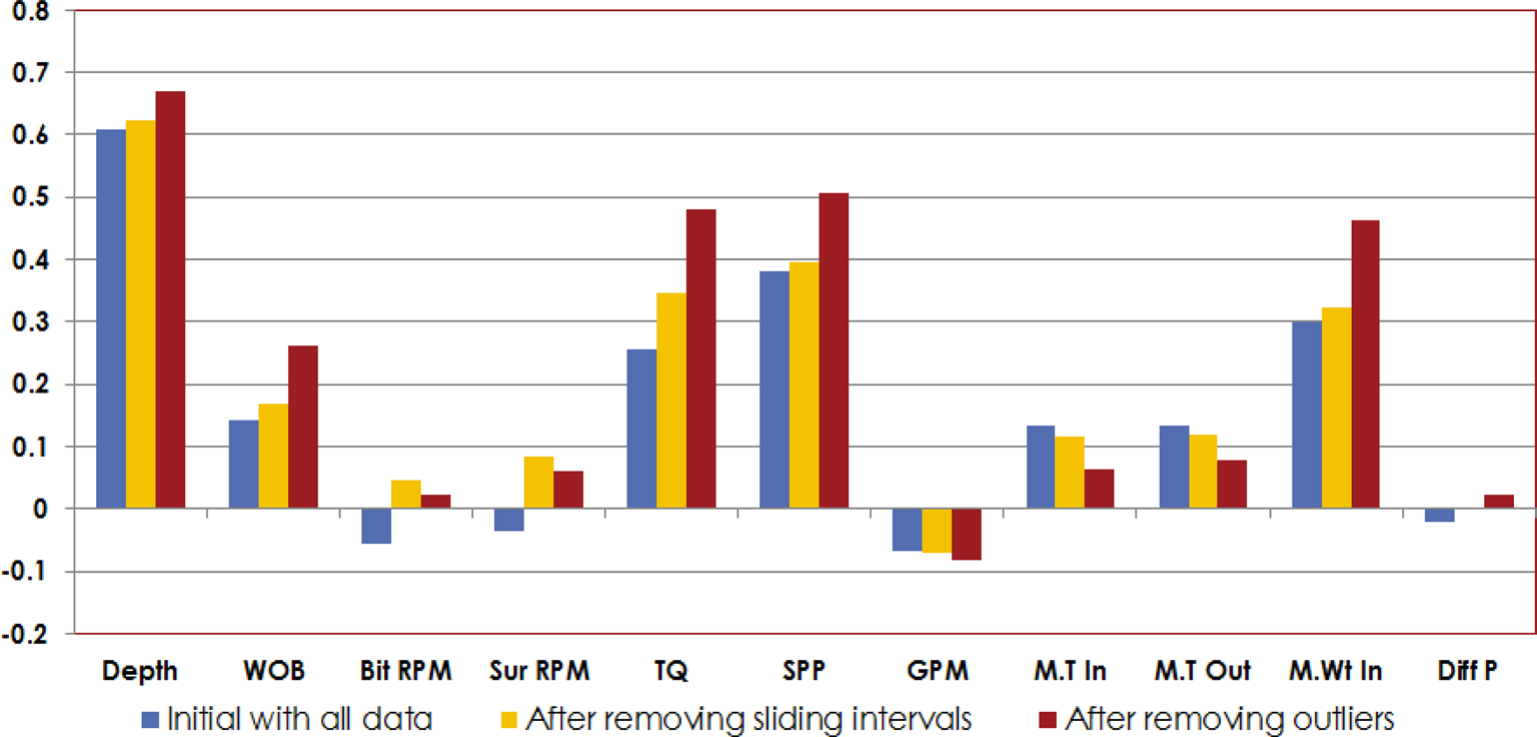
Correlation coefficient (R) between different inputs and the output (GR) before and after data cleaning.

**Figure 4:**
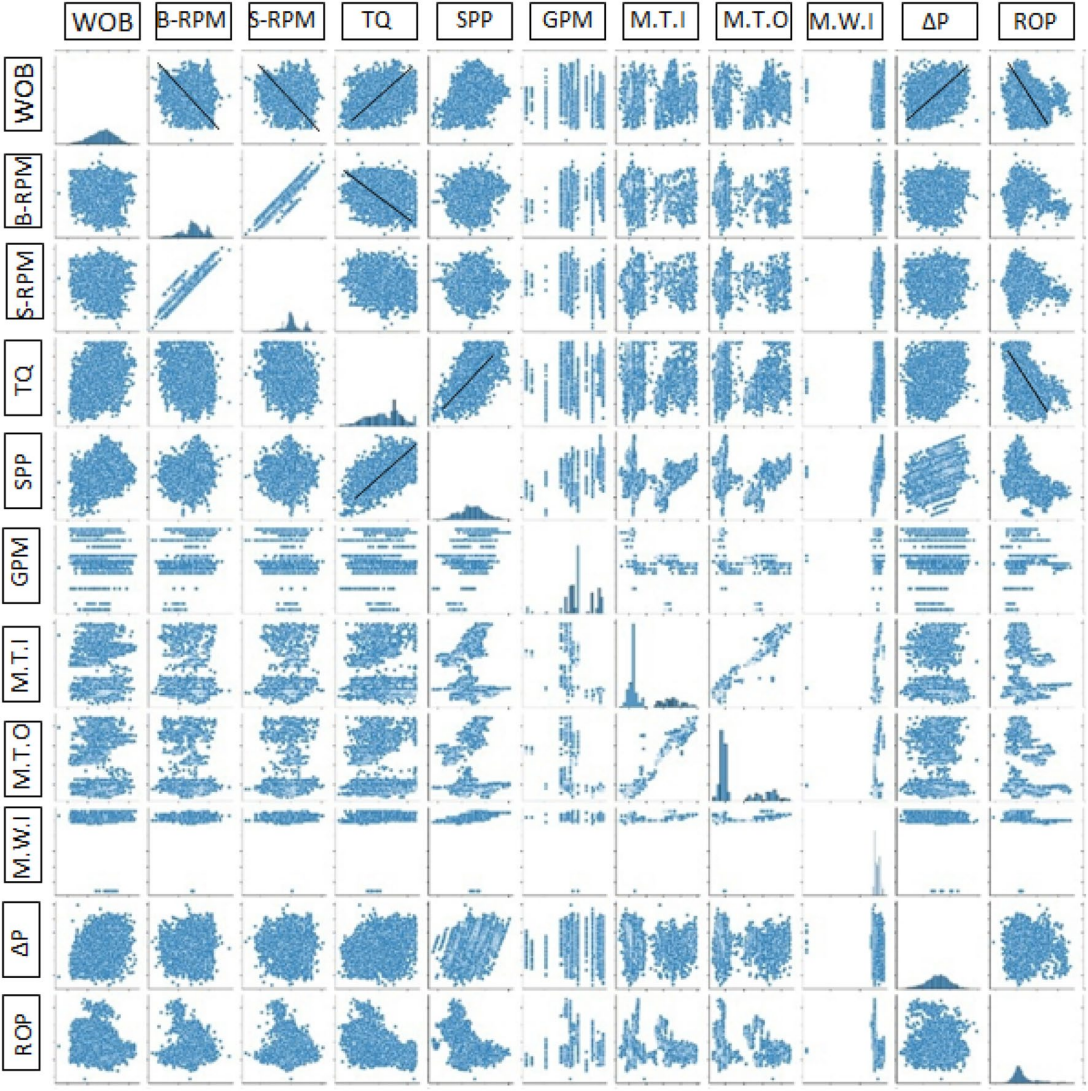
Scatter matrix plot of data features, Where WOB = weight on bit (Klbm), B-RPM = bit rotation (revolution per minute), S-RPM = surface rotation (revolution per minute), TQ = surface torque (Klbf-ft), SPP = standpipe pressure (psi), GPM = flow in (gallon per minute), M.T.I = mud temperature in (deg C), M.T.O = mud temperature out (deg C), M.W.I = mud weight in (ppg,), ΔP = differnentila pressure (psi), ROP = rate of penetration (m/hr).

The collected data for this work was statistically analyzed through performing different statistical analysis as mentioned in Table 1. Figure 2 shows the distribution histogram for each parameter in the dataset after cleaning and filtering. WOB values from 5 to 43 klbf, surface RPM values from 30 to 80 rpm, bit RPM values from 122 to 173 rpm, TQ values from 0.3 to 16.5 kft.lbf, SPP values from 1885 to 3518 psi, GPM values from 717 to 855 gal/ min. M.Wt values from 8.8 to 9.6 ppg, M.T values from 23.4 to 64.5 deg.C, motor differential pressure values from 29 to 595 psi. ROP values from 5 to 88 m/h. Figure 3 shows a scatter matrix plot between the different features to visualize the relationship between each other. The possible trend between the different features is represented by the black line. It’s shown that, there is a positive trend between the different parameters such as WOB, TQ, RPM and motor differential pressure with respect to ROP. Correlation coeffient identity matrix is shown in Table 2 confirming there is good relationship between the different features with each other and with the output. Figure 4, shows the correlation coeffiencnt (R) between the different input features and ROP before data filtering and cleaning, after removing the sliding intervals and after completing data filtering and cleaning. R represents how each parameter is related to the output with a direct or inverse relationship.

**Table 1:** Descriptive statistical summary of the dataset used in this study.

	WOB (Klb)	Bit RPM (Rev/Min)	Surface RPM (Rev/Min)	Torque (lb-ft)	SPP (Psi)	Flow in (GPM)	Mud.T. in (Deg. C)	Mud.T. O (Deg. C)	Mud Wt in (ppg)	Motor ΔP (Psi)	ROP (m/hr)
Minimum	5.8	122.8	30.8	3161.3	1884.2	717.6	14.4	23.4	8.8	29.3	5.0
25%	23.1	122.8	6.9	2780	266	28.7	14.4	28.5	9	253.1	25.3
50%	27.4	145.9	30.7	3161	1884	717.64	21.7	29.4	9.2	313	29.3
75%	31.12	150.1	57.45	8985	2553	797	22.6	46	9.3	366	36.1
Maximum	43.2	173.4	80.6	16,456.1	3518.5	855.0	59.3	64.5	9.6	595.1	88.1
Average	27.0	149.7	60.4	11,095.9	2722.4	811.8	29.3	35.9	9.2	308.0	32.9
STDEV	5.8	7.5	6.9	2780.7	268.1	28.7	11.3	11.3	0.2	87.1	12.9
Skew	− 0.25	− 0.52	− 0.35	− 0.22	− 0.03	− 0.84	1.04	1.06	0.27	− 0.21	1.63

**Table 2:** Correlation coefficient analysis among the input and output features.

	WOB	Bit RPM	Surface RPM	Torque	SPP	Flow in	Mud T. in	Mud T. out	M.Wt. in	Diff pressure	ROP
WOB	1.00	0.03	0.05	0.41	0.28	− 0.03	− 0.06	− 0.05	0.19	0.29	− 0.26
BIT RPM	0.03	1.00	0.91	0.06	0.27	0.38	− 0.12	− 0.08	0.26	0.08	− 0.02
Surface RPM	0.05	0.91	1.00	− 0.04	0.10	− 0.04	0.09	0.11	0.28	0.01	− 0.06
TQ	0.41	0.06	− 0.04	1.00	0.65	0.23	− 0.13	− 0.10	0.42	0.30	− 0.48
SPP	0.28	0.27	0.10	0.65	1.00	0.44	− 0.17	− 0.14	0.70	0.33	− 0.51
GPM	− 0.03	0.38	− 0.04	0.23	0.44	1.00	− 0.49	− 0.43	0.00	0.17	0.08
Mud T. .In	− 0.06	− 0.12	0.09	− 0.13	− 0.17	− 0.49	1.00	0.99	0.17	− 0.10	− 0.06
Mud T. Out	− 0.05	− 0.08	0.11	− 0.10	− 0.14	− 0.43	0.99	1.00	0.20	− 0.09	− 0.08
M.Wt.In	0.19	0.26	0.28	0.42	0.70	0.00	0.17	0.20	1.00	− 0.08	− 0.46
Diff P	0.29	0.08	0.01	0.30	0.33	0.17	− 0.10	− 0.09	− 0.08	1.00	− 0.02
ROP	− 0.26	− 0.02	− 0.06	− 0.48	− 0.51	0.08	− 0.06	− 0.08	− 0.46	− 0.02	1.00

### Model development

The 5800 data points obtained from six wells were divided randomly into two sets of data, one for training and validating the model and the other set for testing the model. The proposed models were developed by employing ANN and ANFIS techniques.

ANN could be considered as ML powerful technique that is capable to find an accurate solution for complex engineering cases and it is built using the principles of neuronal organization. ANN model mainly consists of a combination of connected nodes defined as artificial neurons. Each neutrons connection has the ability to transmit the signal to other neurons. Processing the signal after receiving it is the main function of the artificial neuron. The conventional structure of a neural network mainly contains three types of layers: the first layer for input features, hidden layer(s), and the last layer for output feature ^44^. The input layer will contain the different input features whose have different weights to be connected to the hidden layers(s). The neurons in that layer(s) will perform the required processing on the input features and after that; they transferred to the output layer. The lowest possible error associated with a specified network configuration could be achieved by adjusting each feature weight and the biases as well ^45^.

Using ANN, motorized BHA ROP could be predicted using a developed models based on the drilling parameters, the mud parameters and the motor output as feeding inputs. The criteria for selecting the best network parameters is to achieve the highest R-value with the minimum RMSE and AAPE. The minimum error could be obtained from a model with 4 layers configuration: one for input features (the drilling parameters, the mud weight and temperature and the motor output), 9 neurons for the first hidden layer, 15 neurons for the second hidden layer, and the final layer for the output layer (ROP). The training function of that model was the Levenberg Marquardt algorithm (trainlm) and tan-sigmoidal function was selected as a transfer function for the input layer while a linear function was selected for the output layer.

Another model using ANN was built and trained based on 5 wells and the six unseen well was used for testing. The best network parameters were combined to achieve the highest R-value with the lowest RMSE and AAPE. The minimum error could be obtained from a model with the same layer configuration as the previous model with 10 neurons for the first hidden layer and 16 neurons for the second hidden layer. The training and transfer functions of that model were the same as the previous model.

Similarly, ANFIS model which could be considered as decision making tool was built through adjusting the hyperparameters to achieve the highest model accuracy. The conventional ANFIS structure usually has five layers. The input values are represented in the first layer with their membership function so the common name for this layer is the fuzzification layer. Generating the different rules is associated with the second layer which is commonly known as rule layer. Normalization of the computed strengths is performed through the third layer. The normalized values from the previous layer are stored along with the consequence parameter set in the fourth layer. The final output results are presented into the fifth layer after defuzzification the values obtained from the fourth layer ^46^. The ANFIS developed model input membership function was “gaussmf” and the output membership function was “linear”. The epoch size was set at 250 with 0.4 as cluster radius.

## Results and discussion

### Artificial neural network (ANN)

**Figure 5:**
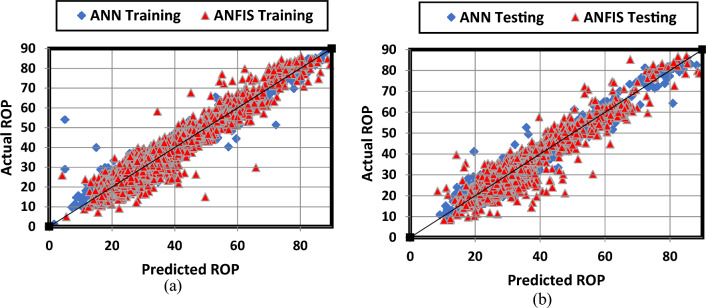
Crossplots for ANN an ANFIS Model; (**a**) Training and validation (4060 point 0.70% of total data) and (**b**) Testing (1020 point = 0.30% of total data).

ANN model proved its validity to predict ROP in a Real-time using the surface drilling parameters, motor output, mud weight and temperature which are measured through a sensor on the mud return line. The RMSE between the real and the model ROP was 2.9 and 3.1 for the training and testing respectively and the correlation coeffient was found 0.97 and 0.97 for the training and testing dataset respectively. Figure 5 shows that most of the data match with the 45° line for the training and testing.

**Figure 6:**
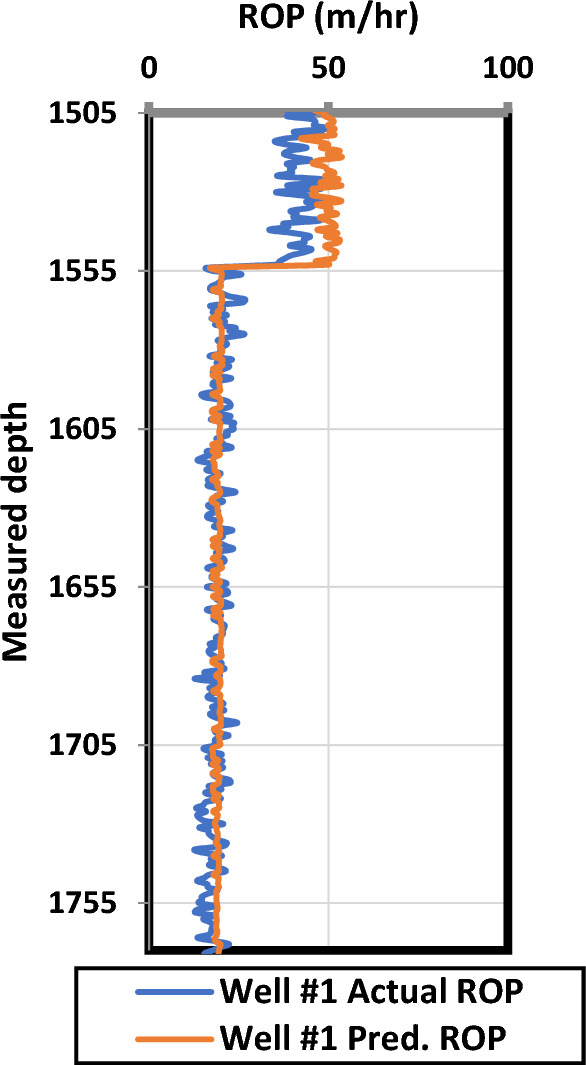
Prediction performance of the developed model (Actual vs. Predicted motor ROP) for the ANN verification process.

Figure 6 represents the predicted ROP against the actual ROP for a complete unseen well using the second developed model that was trained based on five wells and the sixth well was for testing. The RMSE between the actual and the predicted ROP was 2.6 and 5.6 for the training and testing respectively and the correlation coeffient was found 0.97 and 0.96 for the training and testing dataset respectively.

### Adaptive neurofuzzy inference system (ANFIS)

Similarly, ROP values could be predicted based on the drilling parameters, motor output, the mud weight and temperature using the ANFIS model. RMSE between the actual and the predicted ROP was 3.6 and 4.3 for the training and testing respectively and the correlation coeffient was found 0.96 and 0.94 for the training and testing dataset respectively. Figure 5 shows that most of the data align with the 45° line for the training and testing.

**Figure 7:**
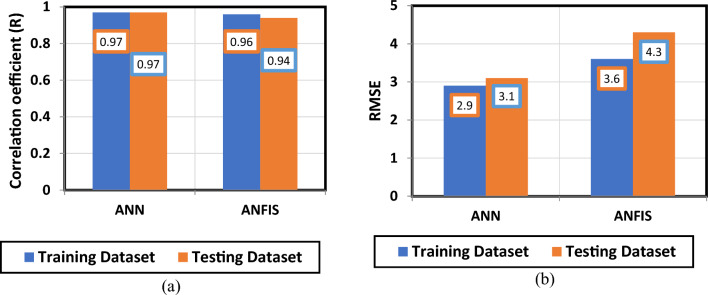
ANN and ANFIS models performance indicators; (**a**) Correlation coefficient, and (**b**) Root mean square error.

A bar chart for the R for ANN and ANFIS models for training and testing datasets is shown in Fig. 7a. The R for the two models is higher than 0.94 for the training and testing datasets. RMSE for both models is represented in Fig. 7b for the training and testing datasets. In general, total AAPE for the training and testing datasets for ANN model was 7.9% and for ANFIS model was 9.1% indicating that ANN model is slightly outperforming ANFIS model but the two models are capable to predict the motorized assembly ROP values accurately.

This study confirms the possibility of predicting ROP for a motorized BHA using the surface drilling parameters, motor output and the mud parameters as well. ROP prediction is very important for well cost estimation and determining the expected AFE. As the drillabilty of the formation is controlled by the lithology, this means when the formation drillabilty decreases, it requires more WOB, high RPM to drill this formation with a reasonable ROP. Both ANN and ANFIS AI models were capable to accurately predict motor BHA ROP in a Real-time which will help for selecting the best drilling parameters to achieve maximum ROP, minimizing the hole exposure time and saving a significant time and cost.

A broad ROP management program is one of the main tools that help the onsite drilling engineer to achieve the optimum ROP. This could be implemented by selecting the optimized ROP-related parameters. Those optimized parameters could be determined based on the developed model by running the developed models to simulate the effect of different configurations of the ROP-related parameters then study their effect on the modeled ROP to end up with the selection of the optimized parameters that are associated with maximum ROP. By doing this, we will have an effective ROP management program. As it’s clearly known this ROP management program is dependent on the BHA type, some important parameters related to downhole motors such as the downhole motors differential pressure and downhole motors rotation output are included in the model inputs to model the motor performance and effect. One of the important applications of this model is to end up with the optimized ROP-related parameters including downhole motor operation parameters.

It should be highlighted that the developed model is based on 12¼” bit. This can be explained as different bit sizes may have different drilling parameters response. So if this model is used with different bit size, this may lead to some errors. The developed model is also based on $${9}{\raise0.7ex\hbox{$5$} \!\mathord{\left/ {\vphantom {5 8}}\right.\kern-0pt} \!\lower0.7ex\hbox{$8$}}$$ “Geoforce motor with 6/7 lobes, 3.5 stages, 0.11 RPG, with1.15 BH and $${12}{\raise0.7ex\hbox{$1$} \!\mathord{\left/ {\vphantom {1 8}}\right.\kern-0pt} \!\lower0.7ex\hbox{$8$}}$$” sleeve so care should be given when using a different down hole motor as it may have different mud motor output in terms of flow rate and differential pressure. It’s recommended to apply the developed models using input parameters within the same range mentioned in Table 1 to have accurate results. The proposed models accuracy level could be improved by including other parameters that affect the ROP such as MSE and this improvement could be included in a future research.

## Conclusions

In this study, two different machine learning techniques were developed, ANN and ANIS, to predict motorized BHA ROP. The developed models used the surface drilling parameters data: WOB, RPM, TQ, SPP, GPM, motor output parameters: motor RPM and motor differential pressure, the mud weight and temperature as feeding inputs to the model. The main conclusions of that research can be highlighted as follows;

- ROP is affected by the drilling parameters and a pressure-related phenomenon known as chip hold-down as well.
- Accurate prediction for the motorized BHA ROP based on the surface drilling parameters, motor output, mud weight and temperature was obtained using ANN and ANFIS models with a correlation coefficient above 0.94 between the actual measured GR values and the model prediction values.
- ANN slightly outperforms ANFIS model but both of them proved their ability to capture most of the changes in ROP and good matching is obtained between the measured ROP and model predicated values.
- Motorized BHA ROP could be optimized by adjusting both the drilling parameters and mud motor output.
- Down hole failures could be detected using the proposed model when the model ROP is overestimated than the actual ROP.

## Data Availability

Due to data privacy, supporting data cannot be made openly available. Contact the corresponding author for further information about the data and conditions for access.

## Abbreviations

AAPE: Average absolute percentage error
AFE: Authorization for expenditure
AI: Artificial intelligence
ANFIS: Adaptive neurofuzzy inference system
ANN: Artificial neural network
BH: Bent housing
BHA: Bottom hole assembly
CCS: Confined compressive strength
GPM: Gallon per minute
HMSE: Hydro-mechanical specific energy
M.T: Mud temperature
M.Wt: Mud weight
MSE: Mechanical specific energy
PDC: Polycrystaline diamon compact
R: Correlation coefficient
RMSE: Root mean square error
ROP: Rate of penetration
RPG: Revolution per gallon
RPM: Revolution per minute
RSS: Rotary steerable system
SPP: Stand pipe pressure
SPP_off BTM_: Off bottom stand pipe pressure
SPP_on BTM_: On bottom stand pipe pressure
SVM: Support vector machine
TFA: Total flow area
TQ: Torque
WOB: Weight on bit
ΔP_MTR_: Motor differential pressure
